# Observations on Tertian and Quotidian Malarial Fevers

**Published:** 1903-03

**Authors:** S. D. Kolozsvary


### OBSERVATIONS ON TERTIAN AND QUOTIDIAN MALARIAL FEVERS.

BY S. D. KOLOZSVARY, M.D.

Although the antipyretic effect of cinchona bark has been known for almost five hundred years, and cinchona preparations have been in use since 1820 as antipyretics and specifics against malaria, attempts have been repeatedly made to replace quinine by various derivatives. The reason for this is the unpleasant, bitter taste of quinine and also its disagreeable by-effects, such as headache, tinnitus, and vomiting, which are obstacles to its employment in many instances.

Several years ago a quinine preparation named saloquinine was sent to the clinic of Professor Purjesz for experimental purposes. Its chemical properties are as follows: Saloquinine is a salicylic acid ester of quinine, and occurs as an odorless and tasteless powder. It is readily soluble in warm alcohol and in benzol, but dissolves with difficulty in ether and cold alcohol. Unfortunately the preparation reached us late in the year, at a time when malarial affections are on the decrease. Moreover, we have observed comparatively few malarial cases in the past year. I first experimented with saloquinine on myself, taking three powders of twelve grains each at intervals of half-hour. The powder is absolutely tasteless, and after taking it I experienced no unpleasant effects of any kind.

In various malarial conditions I administered saloquinine in the same manner as recommended by Bacelli in regard to quinine, that is, three hours before the expected attack a large dose was given within a short time. The drug was administered dry upon the tongue, followed by a swallow of water. This was done in order to determine what the patients thought of its degree of tastelessness.

My experiments relate to the following cases:

CASE I.—P. S., admitted September 19, had suffered since about three weeks from feverishness, and since the past two weeks had had every third day febrile attacks consisting of a rigor followed by sweating and headache. During the interval he felt quite well. Up to September 25th he had three typical tertian attacks with elevation of temperature to 42° C. On the morning of September 25th, examination of the blood revealed the following: Red blood-corpuscles somewhat paler; poikilocytosis; lymphocytosis; numerous quite large tertian parasites in the beginning of sporulation. On this day the patient received three hours before the attack thirty-three grains of saloquinine. The chills failed to appear, and the highest temperature did not exceed 38.8° C. An examination on the following day before the patient left the hospital, which he did against our advice, showed an absence of parasites in spite of careful search.

CASE II.—I. S., fifteen years old, admitted October 18th, had suffered since two months from attacks of chills every third day. Up to October 20th the highest temperature during the paroxysms was 39.4°. The blood contained numerous tertian parasites. On the same day the patient received thirty-five grains of saloquinine three hours before the expected attack. The maximum daily temperature was 36.8° C. Up to October 24th the patient was free from fever and no malarial parasites could be detected in spite of repeated examinations.

CASE III.—V. K., two years old, had been feverish since two weeks. During several days elevations of temperature from 37.4° to 37.9° were noted. The blood contained tertian parasites of two generations in moderate numbers. Later, on the twenty-first day of his stay at the hospital, the fever assumed the course of alternating quotidian type, with exacerbations ranging up to 38.8° C. on odd days and 40.5 to 40.7° C. on even days. Three hours before the febrile paroxysm of the less marked type eight grains of saloquinine were administered, after which the fever changed from a quotidian to a typical tertian character, the more severe paroxysms only persisting, while their intensity was reduced, the fever not exceeding 38.5° to 37.7°. At the end of three days, however, a recurrence took place of a quotidian type; eight grains of saloquinine were administered three hours before the expected rise of temperature; after which the attacks again became tertian, with temperatures of 38.4° to 39.2°. During the interval there was a condition of apyrexia, this corresponding to the day of administration of saloquinine. Repeated administrations of the drug, however, were necessary to cause a complete disappearance of the attack. In this case, therefore, saloquinine acted exactly like small doses of quinine. It destroyed only those tertian parasites which were not in a state of sporulation, while the others were only weakened. The recurrence is an evidence that the parasites which are sporulating were not completely removed under the influence of saloquinine.

INFECTION WITH QUARTAN PARASITES.

CASE I.—J. V., laborer, thirty-one years old, stated that since a month he had had an attack every second day. Up to October 25th four protracted quotidian paroxysms came under observation. An abundance of quartan parasites was present in the blood belonging to three generations. October 29th the patient received three hours before a paroxysm thirty-two grains of saloquinine in the course of one and one half-hours. On the following day the highest temperature was 37.8° C. The patient remained for four days more under our observation, and during this time there was absence of fever.

CASE II.—A. J., laborer, forty-two years old, complained that he had suffered since four weeks from what he regarded as a cold, with obstinate cough and fever. He had no chills. Up to October 29th we observed daily paroxysms with temperatures up to 40° C., the range of temperature corresponding to a typical quotidian intermittent. In the blood were found abundant quartan parasites belonging to three generations. October 29th, three hours before a paroxysm, thirty-two grains of saloquinine were administered within one and one half-hours, and on this day he remained free from fever. The further course of the temperature showed that on each third day, that is on each day when saloquinine was given, apyrexia occurred, while during the two intervening days exacerbations of temperature to 38.7° and 39.9° C. were noticed. Unfortunately the patient withdrew from further observation.

In this case, also, saloquinine acted in somewhat the same way as small doses of quinine in double quartan; that is, that brood of parasites before whose sporulation it was given was destroyed, while the other two broods were only weakened. At the time of his discharge from the hospital we found in the blood quartan parasites belonging to the second generation.

CASE III.—K. P., nineteen years old, shoemaker, was admitted September 21st, with the complaint that he had suffered for two weeks with a fever at night, which was preceded in the afternoon by headaches and vertigo. Up to September 27th we observed four quotidian attacks, with temperatures up to 40.6° C. September 22d, examination of the blood; quartan parasites in considerable number, belonging to three equally well-developed broods of parasites. September 26th, quartan parasites just before sporulation could be detected. On this day the patient was given, three hours before a paroxysm, 32 grains of saloquinine in the course of one and one half-hours. September 27th the blood contained quite a number of quartan parasites on which the effect of quinine was perceptible. The same day the patient left the hospital.

CASE IV.—P. D., thirty-five years old, laborer, admitted January 24th. The patient stated that since five months he had suffered from malarial attacks, which occurred daily during the first three months and every third day in the last two. He had been under medical treatment, and had taken quinine powder, but without any effect upon the attacks. Up to February 2d he had four typical quartan paroxysms, with temperatures up to 40.3°. Examination of the blood revealed numerous quartan parasites belonging to the first generation. February 13th the patient received, at the end of a paroxysm, forty grains of saloquinine in the course of one and one half-hours, after which the attacks disappeared. During the following eight days, which he passed in the hospital, he remained free from fever. Examinations of the blood were made every day, but failed to show any parasites.

### INFECTION WITH CRESCENT-SHAPED PARASITES.

CASE I.—S. G., admitted December 3d, stated that since yesterday he had suffered with headache, vertigo, lassitude, chills and fever. He had never had malaria. Up to December 9 we observed three malignant tertian attacks, with temperatures up to 40.5° C. In the blood were found crescent-shaped parasites in large numbers. In the evening of December 9th, at the end of a paroxysm, during which the temperature had been 40.3° C., the patient received thirty-two grains of saloquinine in the course of an hour and a half. December 10, the highest temperature was 39.4° C. December 11, in the forenoon, saloquinine, thirty-two grains, was administered within one and one half-hours, and during this day there were no exacerbations of temperature; no parasites could be found in the blood. December 12, in the forenoon, the patient again received thirty-two grains of saloquinine, no rise of temperature taking place. Every fifth day he was given twenty-two grains of saloquinine until December 30th, with the result that no marked paroxysm occurred, and up to the present time his health has continued good.

This case appeals to our interest for the reason that at the time the patient, who was employed in the hospital, suffered from his first attack, experiments were in progress with anopheles, which had been infected with crescent-shaped parasites, and of which a few insects escaped. We had to deal here, therefore, with a case of acute malaria, produced by the sting of an anophele infected with crescent-shaped parasites. The circumstance that the acute malignant tertian forms are not readily influenced by quinine is of special importance in judging of the effect of saloquinine.

CASE II.—J. B., aged twenty-seven years, laborer, admitted October 5th, stated that since about five weeks he had daily attacks. October 7th the temperature curve was found to be that of the quotidian intermittent, with temperature up to 39.5° C. Examination of the blood showed crescent-shaped parasites and spheres. October 7th he received in the evening, between 5 and 5:30 and at 6, two doses of eight grains each of saloquinine. October 15th the patient was free from fever. Examination of the blood, made October 10th and 11th, showed crescents and spheres, but no crescent-shaped parasites. The patient left the hospital October 15th.

CASE III.—Mrs. J. K., twenty-two years old, admitted October 20th, stated that since about two weeks, at five o'clock in the afternoon, she had had attacks of fever of an hour's duration. These had remained absent in the last two days, but she was very weak and had headache, and felt hot, especially in the afternoon. Up to November 1st she had three quotidian paroxysms, with temperatures up to 39.1° C. In the blood were found isolated, crescent-shaped parasites and many crescents and spheres. November 1st, the patient received between 11 and 11:30 and at 12, one dose of eight grains and the other of twelve grains of saloquinine. On the following day there was no rise of temperature. Examination of the blood made on November 2d, 3d, and 5th revealed isolated crescents and spheres, but no crescent-shaped parasites.

CASE IV.—J. B., twenty-two years old, domestic, admitted October 24th, complained since thirteen days of general lassitude, and especially headache and pains in the limbs, as well as daily attacks of fever, which lasted from the afternoon until evening. Three years ago she had suffered with chills and fever for three days. Up to November 26th we observed two quotidian paroxysms, with temperature up to 40.2°. In the blood crescent-shaped parasites in considerable number, and isolated crescents, were found. On the same day, between 12:30 and 1 P.M. and at 1:30 P.M., two doses of saloquinine of eight and twelve grains were administered. The maximum temperature on that day was 38.9°. On the following days there was no elevation in temperature. November 30th examination of the blood showed scanty crescents but no crescent-shaped parasites. On the same day the patient left the hospital.

CASE V.—A. S., seventeen years old, apprentice, admitted October 17th, stated that three and one half weeks ago he had chills and fever for a week. Since that time he had felt languid and had headaches. Examination of the blood revealed crescent-shaped parasites and crescents. On the first four to five days the temperature was subfebrile. On the following days the course of the temperature assumed a quotidian character, mounting up to 39.4°. October 29th the patient received, in the course of one and one half-hours, thirty-two grains of saloquinine. On the following days the temperature ranged from 37.5° to 37.7° C., being sometimes below these figures. November 1st examination of the blood showed isolated crescent-shaped parasites and crescents. During the nine following days after the administration of saloquinine a recurrence took place, with temperature of 40° C. The course of the fever resembled the malignant tertian type. After four days the patient was again given, during one and one half-hours, thirty-two grains of saloquinine, after which the temperature only rose a few decimals above the normal. At the end of five days another recurrence ensued, the fever assuming an intermittent quotidian type. November 28th the patient left the hospital. Before his discharge examination of the blood showed a few crescent-shaped parasites. On December 21st he returned, and stated that since five days he had had attacks of chills daily, between two and five P.M. The course of the disease during the following days resembled that of the intermittent quotidian type, with temperatures up to 40°. Saloquinine, thirty-two grains, was at first administered every day, and later every second day. No further elevations of temperature occurred, with the exception of the fourth day, on which it rose to 37.8°. On December 25th and also 28th the blood was found to contain a few crescents, but no crescent-shaped parasites. In spite of this, however, recurrences took place, with temperatures ranging up to 38° to 38.5° on January 1st and January 7th, but these disappeared after the repeated exhibitions of saloquinine. On January 10th, before leaving the hospital, only one or two crescent-shaped parasites could be found in the blood after careful searching.

CASE VI.—Mrs. M. K., twenty-seven years old, admitted October 23d, had suffered since five weeks with malaise, loss of appetite and headaches, and since two weeks had daily attacks of chills, followed by fever. During several days attacks of intermittent quotidian fever, with temperature up to 40.5°, were observed. In the blood were found crescents in moderate numbers, with only a few crescent-shaped parasites. October 25th the patient received, three hours before an attack, within one and one half-hours, thirty-two grains of saloquinine. Nevertheless, the temperature rose to 39° on this day, as well as the next. The patient again received thirty-two grains of the drug in the above mentioned manner. During the following days the temperature remained subfebrile, ranging up to 37.4°. On the fifth day a recurrence ensued, with temperature up to 39.5°. During the further course of the disease we noticed that at each recurrence, which took place on the fifth or seventh day, the fever disappeared after the administration of saloquinine, but returned at the end of five to seven days. From November 3d the patient received each fifth day fifteen grains of sulphate of quinine. This failed to prevent recurrences at first, but later they disappeared completely. After almost two months' residence in the hospital, the patient was discharged, and at that time careful search revealed only isolated crescents and spheres in the blood, but no crescent-shaped parasites.

Before presenting my conclusions I would mention that saloquinine was considered tasteless by all the patients except one; that in none of the cases did it produce unpleasant by-effects, such as headaches or tinnitus. It will be most easy to understand the action of saloquinine by comparing its effects with those of the sulphate of quinine in the various forms of malarial infection. In infection with tertain parasites the attacks disappeared promptly in all instances after the above doses of saloquinine; the temperature became normal and blood examinations undertaken at a later time failed to reveal any parasites. In cases three, in which there was alternating quotidian fever, the temperature curve, after the administration of saloquinine, assumed a tertian character, while after the continued administration of the drug the febrile exacerbations disappeared completely and no parasites were found in the blood. As is generally known, infection with tertian parasites presents the least resistance to the sulphate of quinine. In all those cases, therefore, saloquinine acted in the same way as an efficient dose of quinine. If we group all diseases due to infection with quartan parasites, we will see that the attacks disappeared under the use of saloquinine, and the temperature fluctuated within normal borders with the exception of one case. In this also we noticed a reduction of the temperature from 40.4° to 37.8°, while on the following days it remained subfebrile.

In the blood of patients who had been for some time under treatment (one to one and one half weeks) we were unable to find any parasites at the time of the patient's discharge from the hospital. In other cases which were under observation for only a short time, and in which the examination of the blood showed quartan parasites of several generations, the number of broods was considerably reduced by saloquinine, although not all of them were completely eliminated. In a case in which the patient left the hospital on the day after the administration of saloquinine we found the parasites altered in a manner analogous to that produced by quinine.

Quartan parasites show more resistance towards quinine than tertian parasites, but on the whole possess only slight resisting power and as a rule are destroyed by sufficient doses of quinine. In regard to the effect of saloquinine upon quartan parasites, the same is true as to what we have said in reference to the tertian parasites, that is, the doses of saloquinine acted exactly like smaller doses of quinine.

The greatest resistance toward the sulphate of quinine is presented by the crescent-shaped parasites, and these often make it necessary to continue its use for some time. In infections with crescent-shaped parasites we observed either a permanent lowering of the temperature or a reduction of fever for two to four days. The crescent-shaped parasites disappeared from the blood after the above doses of saloquinine, in the same way as after the customary doses of quinine. It may happen, however, as in the case of quinine, that, even after repeated administration, isolated parasites may be found. The crescents and spheres are as little influenced by saloquinine as by sulphate of quinine. Recurrences are just as likely to take place, and in those of our patients observed for a long time we gained the impression that recurrences ensue with comparative rapidity, often taking place after four to six days, but receding after repeated administration of saloquinine.

I must once more revert to the first case, in which an acute and severe condition was present, which was effectually combated with saloquinine.

It may be said therefore that saloquinine has proved of service as a specific against malaria and has two advantages over quinine: first, its tastelessness, and second, absence of by-effects.

In this connection it may be mentioned that we have also tried saloquinine in a few cases of typhoid fever in doses of twenty grains in alternation with sulphate of quinine. The antipyretic effect is almost equal in both, but the effect is not produced to the extent observed after antipyrin or phenacetin. It is, however, more lasting, so that the temperature frequently does not rise until after the lapse of three or four days. Saloquinine acts somewhat weaker than quinine as an antipyretic.

*Die Heilkunde, September, 1902.*

